# Utilizing a structure-based virtual screening approach to discover potential LSD1 inhibitors

**DOI:** 10.1007/s00432-024-05784-5

**Published:** 2024-05-15

**Authors:** Zhehao Fan, Xiaofeng Liu, Ning Wang, Shiyi Yu, Caili Bi, Yue Si, Xinyue Ling, Chenxu Liu, Jingcheng Wang, Haibo Sun

**Affiliations:** 1 Institute of Translational Medicine, Medical College, Yangzhou University, Jiangyangzhonglu No. 136, Yangzhou, Jiangsu China; 2 Jiangsu Key Laboratory of Experimental & Translational Non-Coding RNA Research, Yangzhou, China; 3 Yangzhou University Affiliated Northern Jiangsu People’s Hospital, Yangzhou, China; 4 Internal Medicine Department, Haian Hospital of Traditional Chinese Medicine, Nantong, China

**Keywords:** LSD1, Inhibitor, Docking, Molecular dynamics simulation

## Abstract

**Background:**

Lysine-specific demethylase 1 (LSD1) is highly expressed in a variety of malignant tumors, rendering it a crucial epigenetic target for anti-tumor therapy. Therefore, the inhibition of LSD1 activity has emerged as a promising innovative therapeutic approach for targeted cancer treatment.

**Methods:**

In our study, we employed innovative structure-based drug design methods to meticulously select compounds from the ZINC15 database. Utilizing virtual docking, we evaluated docking scores and binding modes to identify potential inhibitors. To further validate our findings, we harnessed molecular dynamic simulations and conducted meticulous biochemical experiments to deeply analyze the binding interactions between the protein and compounds.

**Results:**

Our results showcased that ZINC10039815 exhibits an exquisite binding mode with LSD1, fitting perfectly into the active pocket and forming robust interactions with multiple critical residues of the protein.

**Conclusions:**

With its significant inhibitory effect on LSD1 activity, ZINC10039815 emerges as a highly promising candidate for the development of novel LSD1 inhibitors.

## Introduction

**Fig. 1:**
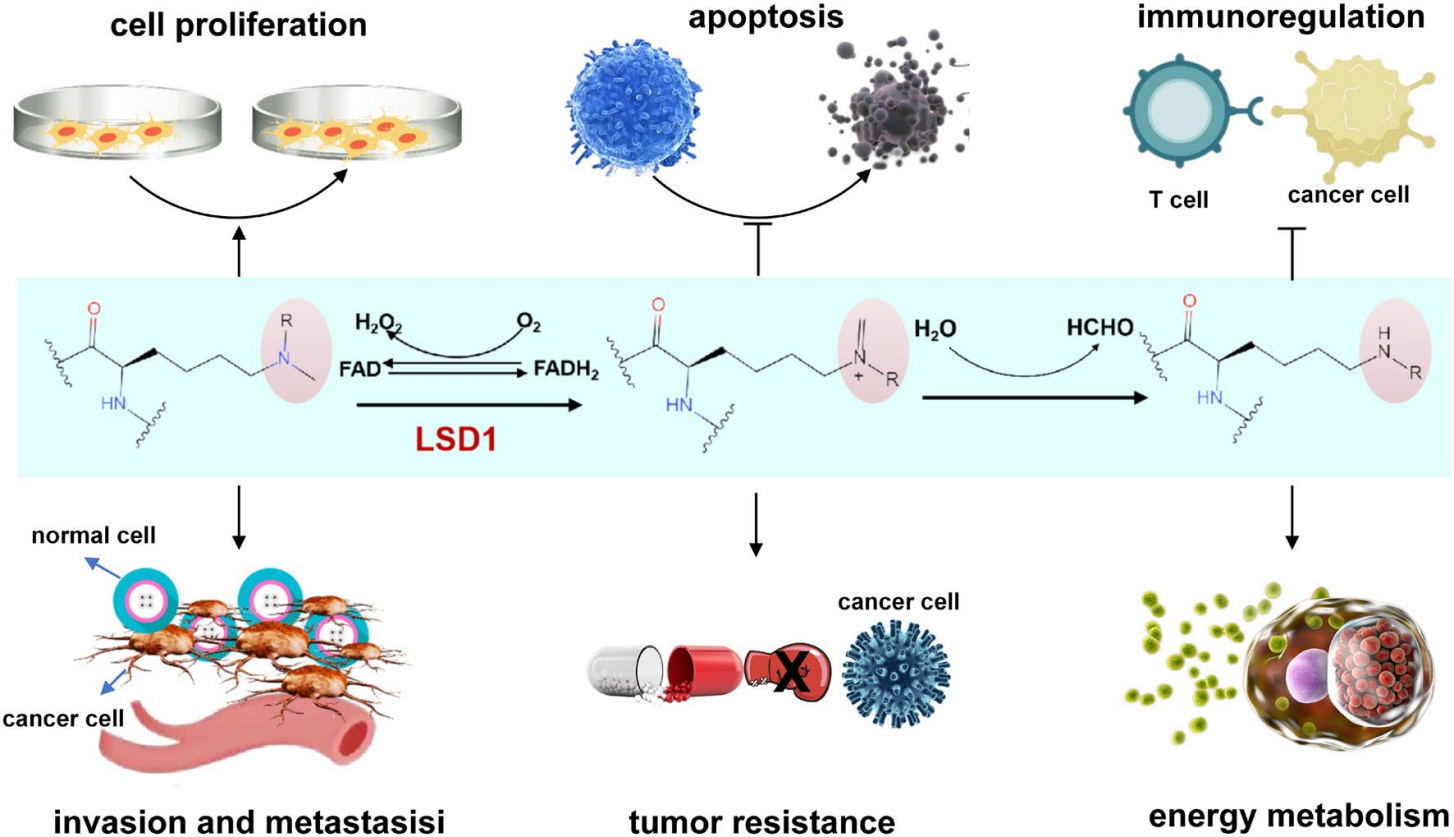
The demethylation mechanism of histone demethylases and their impact on tumor cells

Epigenetic modifications play a pivotal role, not only in governing the physiological development and maintaining stable gene expression in eukaryotic organisms but also in driving the progression of various diseases (Sun et al. 2022). Epigenetic markers, encompassing histone modifications, DNA methylation, non-coding RNAs, and chromatin conformation, wield profound influence over the intricate regulation of gene expression. Through their intricate interplay, they intricately shape cellular function, orchestrate tissue development, and even modulate the risk of various diseases (Hogg et al. 2020). Histone modifications, including but not limited to methylation, acetylation, phosphorylation, and ubiquitination, exemplify a rich and vital array of epigenetic regulatory mechanisms. These modifications intricately govern the intricate orchestration of gene expression, and their dysregulation is a common hallmark of cancer. Consequently, they contribute significantly to the pathological processes involved in tumor initiation and progression (Audia and Campbell 2016). Histone methylation stands out as one of the most prevalent and fundamental modifications, primarily targeting arginine and lysine residues of histones H3 and H4. Its pivotal role in governing gene expression, cell cycle control, genome stability, and the maintenance of nuclear structure is indispensable to the intricate machinery of cellular function (Black et al. 2012). LSD1, also known as KDM1A/AOF2, holds the distinction of being the pioneering specific demethylase, responsible for the removal of methyl groups from histones. Its discovery has been transformative, providing unequivocal evidence that histone methylation is under the precise control of both histone methyltransferases and demethylases, thereby ensuring a delicate and dynamic balance. This groundbreaking finding has shattered the conventional belief that histone methylation is an irreversible process, revealing the intricate regulatory mechanisms governing this crucial epigenetic modification (Fang et al. 2019). In the realm of epigenetic regulation, LSD1 exerts its specific action by targeting mono- and dimethylation of H3K4 and H3K9 through a precise flavin adenine dinucleotide (FAD)-dependent amine oxidation reaction (Fig. 1). This elegant mechanism enables LSD1 to delicately modulate the gene activation potential associated with H3K4 methylation and the transcriptional repression role commonly linked with H3K9 methylation. Such intricate control underscores the significance of LSD1 in maintaining the delicate balance of gene expression in cellular processes (Gu et al. 2020). LSD1 assumes a critical role in a wide spectrum of physiological and pathological processes, encompassing pivotal contributions to embryonic development, neural development, cell cycle regulation, chromatin remodeling, and the intricate orchestration of gene expression. Its multifaceted functions underscore the profound impact of LSD1 in shaping the intricate landscape of cellular dynamics and molecular events.

Emerging research has shed light on an additional facet of LSD1's influence, revealing its capability to modulate the expression of oncogenes, thus fostering the proliferation, invasion, and metastasis of tumor cells. These recent findings further underscore the complex and diverse role of LSD1 in cancer development (Fig. 1). LSD1 emerges as a pivotal player in various tumor diseases, including breast cancer, colorectal cancer, lung cancer, and gastric cancer, where its elevated expression correlates with tumor grade, malignancy, and prognosis. Its regulatory effects on crucial factors such as p53, ERα, and AR contribute significantly to tumor progression (Huang et al. 2007; He et al. 2020). LSD1's impact extends beyond transcriptional regulation, as it modulates post-transcriptional processes, influencing the expression of tumor-related genes like PTEN, thereby fostering tumor cell proliferation and invasion (Ma et al. 2022). Furthermore, LSD1's influence extends to tumor stem cells, regulating their self-renewal and proliferation, thereby driving tumor growth and development. Its intricate association with diverse cellular functions, such as epithelial-mesenchymal transition, cell proliferation, differentiation, stem cell biology, and malignant transformation, showcases the multifaceted role of LSD1 in cancer pathogenesis (Dong et al. 2022). Remarkably, targeting LSD1 has shown promise in enhancing anti-tumor immunity and inhibiting checkpoints, establishing it as an essential target for anti-cancer drug development (Sheng et al. 2018). These cumulative findings solidify LSD1's prominence as a significant therapeutic target in the quest for effective anti-cancer treatments.

Presently, extensive research is dedicated to LSD1 inhibitors, and several of them have advanced to clinical trials, including TCP, GSK2879552, IMG-7289, ORY1001, SP-2577, and CC-9001 (Song et al. 2022). While these inhibitors demonstrate some level of efficacy against LSD1, they also display limitations in terms of selectivity and pharmacological properties (Fang et al. 2019). Consequently, the pursuit of highly selective, innovative, and potent LSD1 inhibitors remains a promising avenue in the quest for more effective and targeted cancer treatments.

**Fig. 2:**
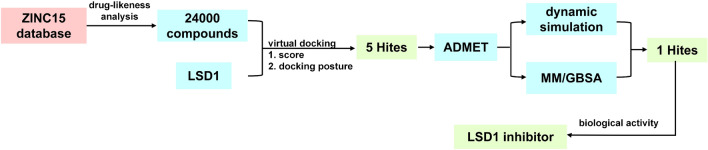
The screening process of LSD1 inhibitors

Structure-based virtual screening stands as a rapid, efficient, and cost-effective drug development approach widely employed in the field of drug research and development (Ballante et al. 2021). Leveraging the power of computer simulation technology, this method enables the screening and prediction of compounds with robust binding affinity to known molecular targets, offering a gateway to potential therapeutic drugs. The LSD1 inhibitor SP-2577, identified through structure-based virtual screening, has received approval from the Food and Drug Administration (FDA) for treating Ewing's sarcoma (Shen et al. 2024). Additionally, CC-90011 has progressed into clinical trials for the treatment of prostate cancer, small cell lung cancer, and other indications (Noce et al. 2023). Therefore, in our study, we used the potential of structure-based virtual screening to identify potential inhibitors of LSD1 (Fig. 2). Encouragingly, our results highlight ZINC10039815 as a novel and promising LSD1 inhibitor, presenting valuable structural leads for the development of more potent and effective LSD1 inhibitors.

## Materials and methods

### Virtual docking

In this study, we used a virtual screening method based on structure to find candidate inhibitors that could target the protein LSD1. In order to provide a solid foundation for our virtual screening study, the crystal structure of LSD1 (PDB ID: 2Z5U) was obtained from the RCSB protein database (https://www.rcsb.org/) and painstakingly constructed by removing water molecules and natural ligands using PyMOL. The receptor file was preprocessed with Autodock to increase the precision of our docking computations. Polar hydrogen atoms were assigned, Gasteiger charges were computed, and the protein structure underwent energy minimization and geometric optimization. The LSD1 active site was then surrounded by a grid box with an XYZ grid size of 100, 100, and 100, precisely placed at coordinates 33.298, 52.145, and 47.373 for the molecular docking procedure (Sorna et al. 2013). These thorough preparations ensured an accurate and reliable docking analysis, setting the stage for our virtual screening approach. Subsequently, we harnessed the power of the USR-VS online tools (http://usr.marseille.inserm.fr/) to perform an exhaustive screening of the ZINC15 database (https://zinc15.docking.org/) (Li et al. 2016). We mainly screened compounds with similarities to LSD1's reversible inhibition of SP-2577, CC-90011 and other potential reversible inhibitors reported in the existing literature, and finally formed a database of 24,000 compounds for further screening (Dai et al. 2021). Before conducting docking, the selected compounds underwent hydrogenation and energy minimization processes in PyRx, ensuring their structural integrity and optimal conformation for subsequent analyses. Molecular docking was then executed using PyRx Autodock Vina, with the LSD1 protein held rigid and the ligand molecules permitted up to 100 degrees of freedom, allowing for a degree of flexibility (Soudani et al. 2021). The docking scores obtained from virtual screening were leveraged to rank and prioritize the compounds based on their potential to interact effectively with LSD1. Only the compounds exhibiting the most robust binding affinity and promising prospects for impacting LSD1 activity were selected for further comprehensive analysis.

### ADMET prediction

We also predict the ADMET properties (absorption, distribution, metabolism, excretion and toxicity) of potential inhibitors of LSD1. To accomplish this, we employed two web servers, namely Swissadme (http://www.swissadme.ch/index.php#top) (Daina et al. 2017) and ADMETlab (admetmesh.scbdd.com/service/) (Xiong et al. 2021), for a comprehensive analysis of these essential properties. We subjected these compounds to the ADMET prediction tools available on Swissadme and ADMETlab. These web servers have proven to be valuable resources in the field of drug development as they provide valuable insights into the pharmacokinetic and toxicological properties of candidate compounds. Swissadme is a well-established web server that offers a range of ADMET-related predictions, including blood–brain barrier permeability, intestinal absorption, and CYP450-mediated metabolism, among others. By utilizing Swissadme, we gained critical information on the ability of our LSD1 inhibitors to be absorbed and distributed within the body, as well as their potential interactions with key metabolic enzymes. ADMETlab, on the other hand, complements the analysis by providing a comprehensive assessment of various toxicity endpoints. This includes predicting potential hepatotoxicity, cardiotoxicity, and other adverse effects, which are crucial aspects to consider in the drug development process.

### Molecular dynamics simulations and MM/PBSA

Molecular dynamics simulations were performed using GROMACS 2022 with Amber 19SB force fields (Kutzner et al. 2022). The optimal binding conformation of ligands obtained from the previous docking study was hydrogenated using PyMOL. The ligand topologies were generated using the ACPYPE server, and they were combined with the protein topology generated by the pdb2gmx module of GROMACS to create the topology files for the ligand–protein complexes. Each complex was placed at the center of a cubic box with an edge length of 1 nm from the protein surface. Water molecules were added using the TIP3P model, and appropriate amounts of Na^+^ and Cl^–^ ions were added to neutralize any additional charge in the system. The energy minimization was performed using the steepest descent method algorithm with 50,000 steps. Next, a 50 ns equilibration run was conducted in the NVT (constant number of particles, volume, and temperature) and NPT (constant number of particles, pressure, and temperature) ensembles at a temperature of 300 K and a pressure of 1 bar. After the equilibration, production molecular dynamics simulations were conducted for 100 ns at constant temperature and pressure. During these simulations, the trajectories of the complexes were recorded for further analysis. Root mean square deviation (RMSD) and root mean square fluctuation (RMSF) analyses were performed using the RMS module of GROMACS to study the stability and flexibility of the ligand–protein complexes over the course of the simulations. Additionally, hydrogen bond interactions between the ligands and the protein were analyzed and recorded (Xu et al. 2022). To visualize the structures and analyze the trajectories, the HeroMDanalysis tool was utilized, which offers comprehensive visualization and analysis options for molecular dynamics simulations. Based on the completed molecular dynamics simulations, the binding energy of each complex was calculated using gmx_MM/PBSA. This method utilizes molecular mechanics/Poisson-Boltzmann surface area (MM/PBSA) calculations to estimate the binding free energy between the ligand and the protein, providing valuable insights into the ligand's binding affinity (Valdés-Tresanco et al. 2021).

### Density functional theory calculation

The electron density refers to the distribution of electrons within a compound, indicating the number of electrons per unit volume and their positions relative to the atomic nuclei. This electron distribution ultimately determines the ground state energy of the compound. Density Functional Theory (DFT) methods provide a powerful approach to connect the electron density with the ground state electronic energy, enabling accurate and efficient evaluation of various properties of the compound. In this study, we conducted DFT calculations using the B3LYP functional correlation along with the 6-31G* basis set (Georgieva et al. 2017). With the DFT calculations completed, we were able to obtain information on the compound's frontier orbitals, such as the Highest Occupied Molecular Orbital (HOMO) and Lowest Unoccupied Molecular Orbital (LUMO). These frontier orbitals provide insights into the compound's electronic transitions and its potential reactivity. Furthermore, the electrostatic potential map obtained from the DFT calculations helps visualize the distribution of electron densities and charges within the compound. This map is valuable in understanding the compound's interactions with other molecules or surfaces, as it highlights regions of high and low electron density.

### Buried surface area (BSA)

Molecular interactions play a crucial role in determining the stability and specificity of protein–ligand complexes. Understanding the physical and chemical properties of the binding interface and the complementary shapes involved is essential for gaining insights into the binding affinity and interaction mechanisms. One key parameter used to characterize protein–ligand interactions is the BSA. BSA measures the amount of surface area buried when a protein–ligand complex is formed. It represents the region of the protein that becomes inaccessible to the solvent upon ligand binding. Calculating BSA provides valuable information about the extent of the interaction between the ligand and the protein, and it correlates with the binding strength and specificity. In this study, we employed the Shrake–Rupley algorithm tool to calculate the BSA between the ligand and LSD1. The Shrake–Rupley algorithm is commonly used to determine the accessible surface area of molecules, and it provides a reliable method to quantify the buried surface area in protein–ligand complexes (Ribeiro et al. 2019).

### Protein purification

1 μl (100 ng/μl) of the Pet15b-LSD1 plasmid was added to Escherichia coli BL21 (DE3) and gently mixed by tapping the tube. The mixture was then left on ice for 30 min and subsequently incubated at 42 °C for 90 s, followed by returning to ice for 3 min. Next, 1 ml of Luria–Bertani (LB) liquid medium was added to the centrifuge tube, and the culture was incubated at 37 °C and 180 rpm for 1 h. Afterward, the culture was centrifuged at 5000 rpm for 5 min, and 100 μl of the supernatant was evenly spread on an LB solid culture plate. The plate was then incubated at 37 °C overnight to allow colony formation. Subsequently, a single colony was picked and inoculated into 5 ml of LB liquid medium supplemented with 5 μl of ampicillin. The inoculated culture was shaken overnight at 37 °C and 180 rpm. Next, 5 ml of the overnight bacterial culture was added to 500 ml of LB medium containing 500 μl of ampicillin, and the culture was shaken at 37 °C and 220 rpm until the optical density (OD) reached 0.6–0.8. Add 1 ml of IPTG (100 mM) to the culture and further shake for 4 h at 37 °C and 220 rpm to induce protein expression, discard the supernatant, add 5 mL loading buffer to the bacterial pellet, break the bacteria with ultrasound, mix the sample gently with a shaker for 60 min, centrifuge at 4 °C at 12,000 rpm for 30 min, aspirate the supernatant into a clean container, and discard the pellet. The supernatant samples were placed on the His-Tag affinity chromatography at a flow rate of 10–15 mL/h, the effluent was collected, and the column was washed with washing buffer (100 mM NaH_2_PO_4_, 10 mM Tris, 8 M Urea, pH 6.3) at a flow rate of 10–15 mL/h, and the column was washed with elution Buffer (100 mM NaH_2_PO_4_, 10 mMTris, 8 M Urea, 500 mM Imidazole). The eluted protein was subsequently dialyzed into 1 × PBS buffer. The protein was then concentrated to the appropriate volume by freeze-drying, and the purity of the purified protein was verified by wstern blot and SDS-PAGE analysis (Shi et al. 2004).

### Biochemical assay

The LSD1 inhibitor SP-2577 received approval from the FDA as an orphan drug for the clinical treatment of Ewing's sarcoma in 2019 (Shen et al. 2024). Consequently, we selected it as the control group for biochemical assay. The enzyme activity of LSD1 was assessed in 96-well plates with a total volume of 100 μl. Compound ZINC10039815 (ChemBridge Economical) selected from the screening, as well as SP-2577, were subjected to continuous twofold dilutions in the range of 0–200 nM. Subsequently, each compound was incubated with 7.5 μg of LSD1 and 5 μg of FAD (Beyotime Biotechnology, Shanghai, China) at room temperature for 15 min. Following the incubation, the substrate H3K4me2 (Dechi, Shanghai, China) 3 μg per well was added to each well, and the plate was further incubated at 37 °C for 30 min to allow the enzymatic reaction to take place. To initiate the detection process, 1 μl of horseradish peroxidase (HRP) (Beyotime Biotechnology, Shanghai, China) and 1 μl of Amplex Red reagent (Beyotime Biotechnology, Shanghai, China) were added to each well. The plate was then incubated at room temperature in the dark for an additional 5 min to allow the enzymatic reaction to proceed. After the incubation period, the fluorescence intensity was measured using a fluorescence spectrophotometer at an excitation wavelength of 530 nm and an emission wavelength of 590 nm. The recorded fluorescence values were then utilized to calculate the corresponding inhibition rate for each compound tested (Maes et al. 2018).

## Result

### Selecting LSD1 inhibitors

**Fig. 3:**
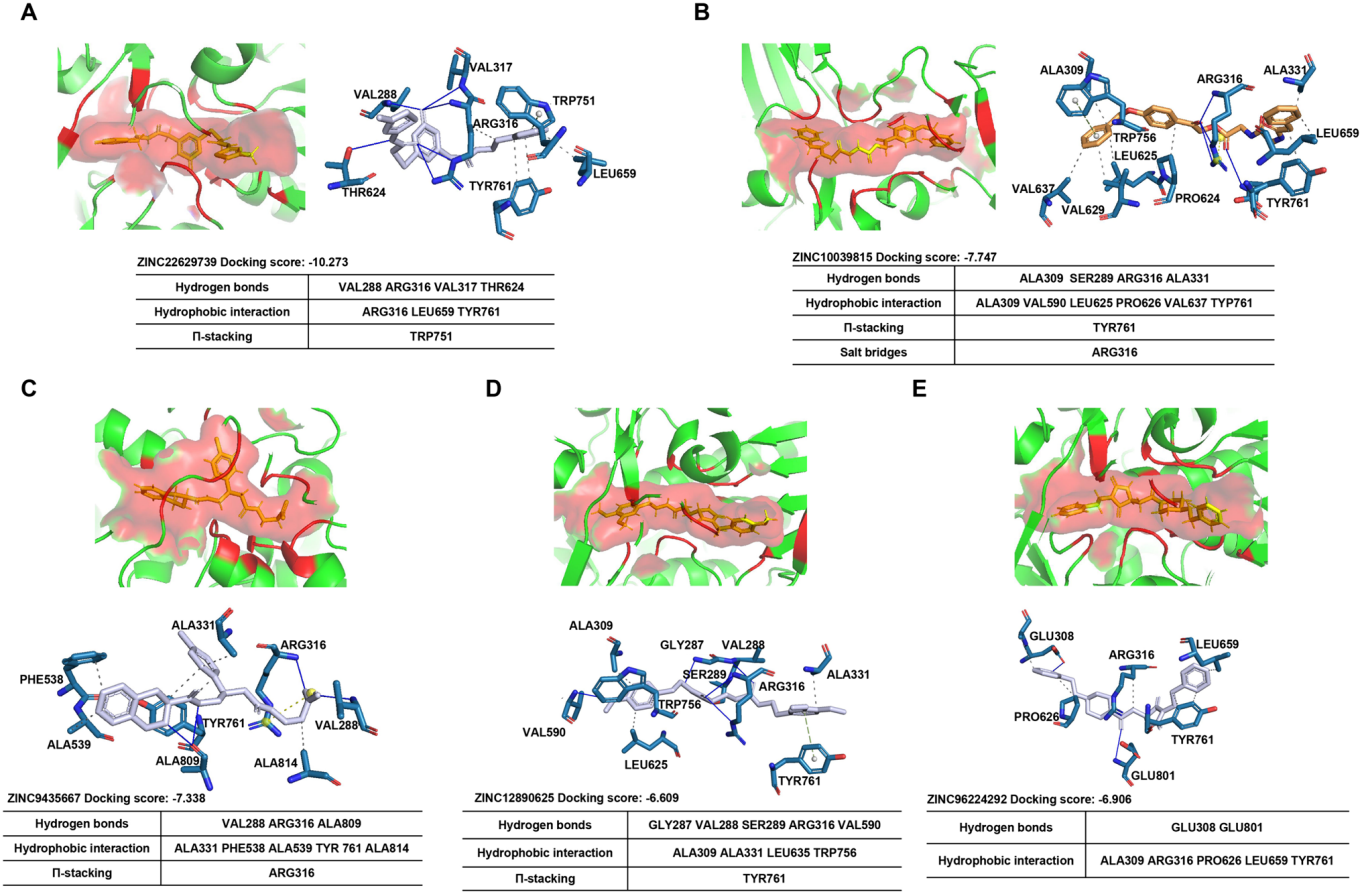
Docking scores with LSD1 protein ranked in the top five compounds and interactions between compounds and complexes. **A** The docking result between ZINC22629739 and LSD1. B The docking result between ZINC10039815 and LSD1. **C** The docking result between ZINC9435667 and LSD1. **D** The docking result between ZINC12890625 and LSD1. **E** The docking result between ZINC96224292 and LSD1

LSD1 (lysine-specific histone demethylase 1) is composed of 852 amino acids. Its crystal structure reveals three main components: the N-terminal SWIRM (Swi3p/Rsc8p/Moira) domain, the C-terminal amine oxidase-like (AOL) domain, and the centrally located tower domain. The AOL domain features a spacious and highly negatively charged substrate-binding pocket, which functions as the catalytically active center of LSD1 and accommodates the N-terminal tail of histone H3 (Shi et al. 2004). To identify potential LSD1 inhibitors, we used the AOL domain as a docking box for the receptor. This led us to compile a database of 24,000 compounds for further analysis. Subsequently, we conducted virtual screening of the selected proteins and small molecules using PyRx software. The compounds were ranked based on docking scores and docking postures, and the top five compounds were identified. The docking score of ZINC22629739 with LSD1 was – 10.273, and it formed hydrogen bond interactions with amino acids at positions 288, 316, 317, and 624 of LSD1, as well as a π-π interaction with the amino acid at position 751 (Fig. 3A). ZINC10039815 had a docking score of -7.747 with LSD1 and formed hydrogen bonds with amino acids at positions 309, 289, 316, and 331 of LSD1. It also formed a π-π interaction with the amino acid at position 761 and a salt bridge with the amino acid at position 316 (Fig. 3B). The docking score of ZINC9435567 with LSD1 was -7.338, and it formed hydrogen bonds with amino acids at positions 288, 316, and 809 of LSD1, as well as π–π interactions with the amino acid at position 316 (Fig. 3C). ZINC12890625 had a docking score of – 6.609 with LSD1, forming hydrogen bond interactions with amino acids at positions 287, 288, 289, 316, and 590 of LSD1, and a π–π interaction with the amino acid at position 761 (Fig. 3D). ZINC96224292 had a docking score of – 6.906 with LSD1 and formed hydrogen bond interactions with amino acids at positions 308 and 801 of LSD1 (Fig. 3E).

### ADMET

Poor pharmacokinetics and toxicity of candidate compounds are significant factors contributing to drug development failures. Early assessment of a ADMET properties can help mitigate risks during drug development (Ferreira and Andricopulo 2019). The ADMET predictions for the five compounds are as follows: All five compounds were predicted to have above-medium solubility. Except for ZINC12890625, all compounds showed good intestinal absorption, indicating favorable absorption potential. Four compounds, excluding ZINC12890625, were not predicted to inhibit CYP2D6, suggesting normal metabolic behavior. ZINC22629739 was predicted to have potential blood–brain barrier permeability, indicating its ability to cross the blood–brain barrier. ZINC10039815, ZINC22629739, and ZINC96224292 were predicted to have some level of hepatotoxicity, indicating possible adverse effects on the liver (Table 1). These ADMET predictions provide crucial insights into the potential pharmacokinetic and toxicological properties of the compounds. Further investigation and optimization of these compounds can help mitigate any pharmacokinetic or toxicity-related issues and increase their chances of success in drug development.

**Table 1:** Properties of Compounds

Compound	ADMET						mm/gbsa (kcal/mol)	Energy		△E = LUMO–HOMO (eV)
	Water solubility	GI absorption	Druglik-eness	BBB	CYP2D6	Hepatoxic		LUMO (Ha)	HOMO (Ha)	
ZINC10039815	Moderately soluble	High	Yes	No	No	Yes	– 89.55	– 0.103456	– 0.16034	1.547870524
ZINC22629739	Soluble	High	Yes	Yes	No	Yes	– 52.25	– 0.094908	– 0.196696	2.769753268
ZINC12890625	Soluble	Low	Yes	No	Yes	No	– 85.06	– 0.075195	– 0.165758	2.464309793
ZINC96224292	Soluble	High	Yes	No	No	Yes	– 73.26	– 0.062747	– 0.166433	2.821399746
ZINC9435667	Very soluble	High	Yes	No	No	No	– 54.69	– 0.118056	– 0.176208	1.582374072

### Analyzing the complexes obtained from docking

**Fig. 4:**
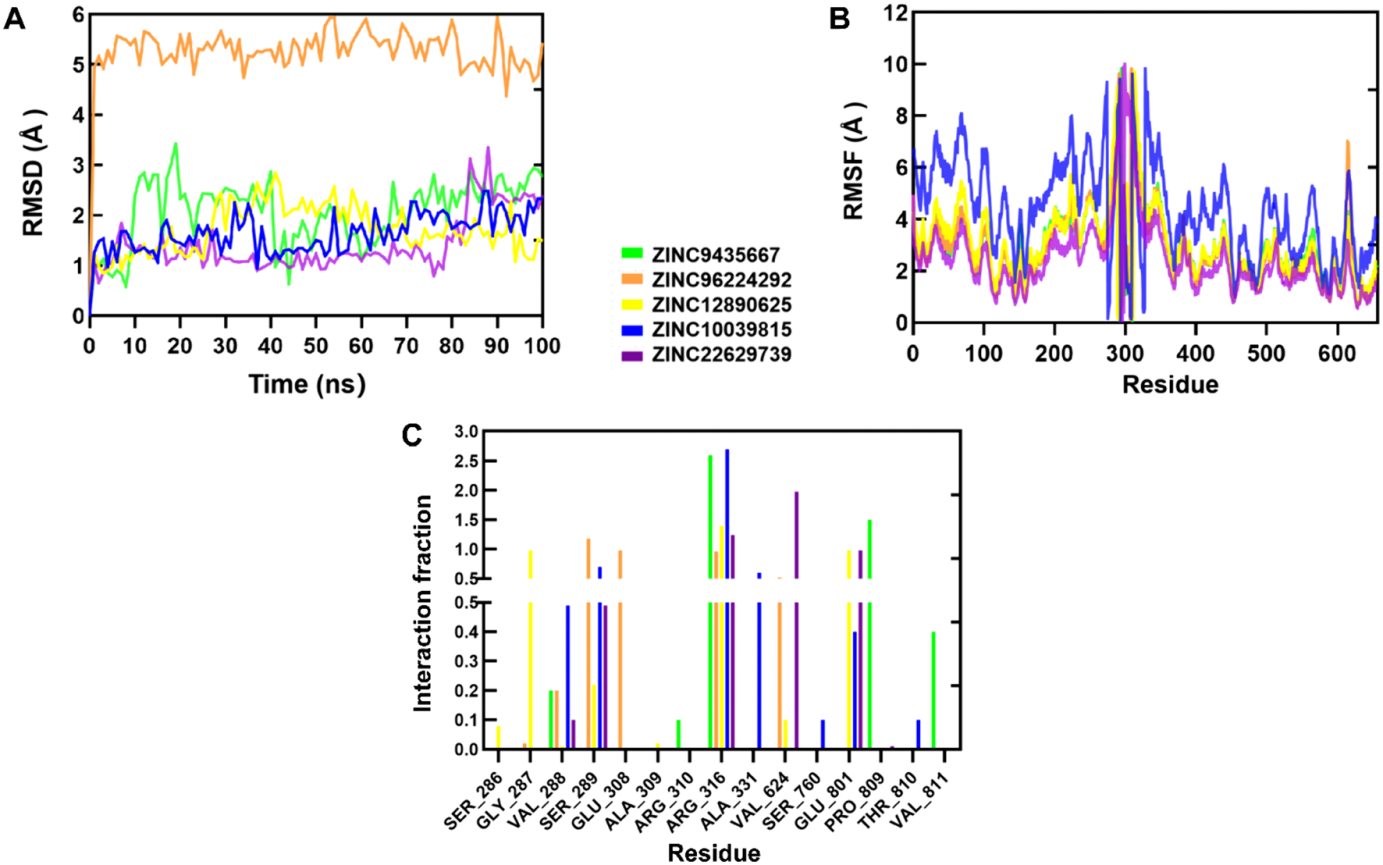
Molecular dynamics simulation results of five compounds with LSD1 protein. **A** RMSD. **B** RMSF. **C** Hydrogen bond interaction analysis

The study aimed to simulate the dynamics of LSD1 in the presence of ligands using the compound and LSD1 docking complex to explore the stability of the protein–ligand docking complex. Molecular dynamics (MD) simulations were utilized to investigate the interactions between the five screened compounds and the LSD1 protein over a 100 ns timeframe. GROMACS was the chosen software for the analysis. The root-mean-square deviation (RMSD) was employed as an important parameter to estimate the time required for the complex to reach structural equilibrium and to assess molecular conformational changes. In Fig. 4A, the RMSD of ZINC9435667 compound fluctuated around 5 Å, while RMSD values of the other compounds fluctuated within 3 Å. This suggests that the structure of the complex exhibits a certain level of stability. Notably, the complex formed by ZINC10039815 and the LSD1 protein gradually stabilized at around 2 Å after 50 ns. Figure 4B demonstrated that the amino acids near the 300th residue of the simulated LSD1 protein exhibited significant fluctuations throughout the simulation process, while the remaining amino acids remained relatively stable. This observation indicates that the overall protein system is relatively stable, and there might be additional binding sites near the 300th residue. After dynamic simulation, hydrogen bond interactions were analyzed, revealing that the five compounds formed hydrogen bonds with amino acids surrounding position 300 of the LSD1 protein. Specifically, ZINC10039815 binds to hydrophobic pockets of LSD1, forming relatively numerous and stable hydrogen bond interactions with amino acids such as GLY288, SER289, ARG316, and ALA331. Moreover, ZINC10039815 also establishes stable hydrogen bonds with amino acids GLU801 and THR810 in the hydrophilic pocket of LSD1. Additionally, ZINC10039815 forms a hydrogen bond with SER760 of LSD1 (Fig. 4C). Based on the analysis of MD simulation results, ZINC10039815 exhibits greater capacity to stabilize the structure of LSD1 compared to the other five compounds, suggesting its potential as a lead compound for LSD1 inhibition.

Binding free energy represents the sum of all non-bonding interactions in a molecular complex. In this study, the MM/PBSA method was utilized to estimate the binding free energy of five compounds and their interactions with LSD1. The interaction energy during the last 5 ns of the MD trajectory, including van der Waals force, polar solvation energy, electrostatic energy, and other relevant factors, was calculated. As shown in Table 1, compound ZINC10039815 demonstrated a higher binding affinity with LSD1, with a binding free energy of -89.55 kcal/mol.

The frontier molecular orbital of a molecule plays a crucial role in determining its interactions with other molecules. Specifically, the HOMO represents the outermost orbital electron and acts as the electron donor, while the LUMO is the innermost orbital and acts as the electron acceptor. The energy gap between the HOMO and LUMO orbitals, known as the energy gap, is a critical parameter for predicting the molecular stability and evaluating the chemical reactivity and kinetic stability of molecules (Aziz et al. 2022). A smaller energy gap indicates stronger chemical reactivity, which can lead to greater instability in the kinetics of the molecule. In Table 1, it is observed that the compound ZINC10039815 exhibits the smallest energy gap, measuring 1.547870524. This suggests that ZINC10039815 is chemically more reactive compared to the other compounds studied. Due to its favorable characteristics, ZINC10039815 is considered a more promising LSD1 inhibitor, as it is likely to form strong interactions with the target protein and potentially exhibit higher inhibitory activity against LSD1. However, further experimental studies and validation are necessary to confirm its effectiveness as an LSD1 inhibitor and its potential as a therapeutic agent.

### Compound ZINC10039815

**Fig. 5:**
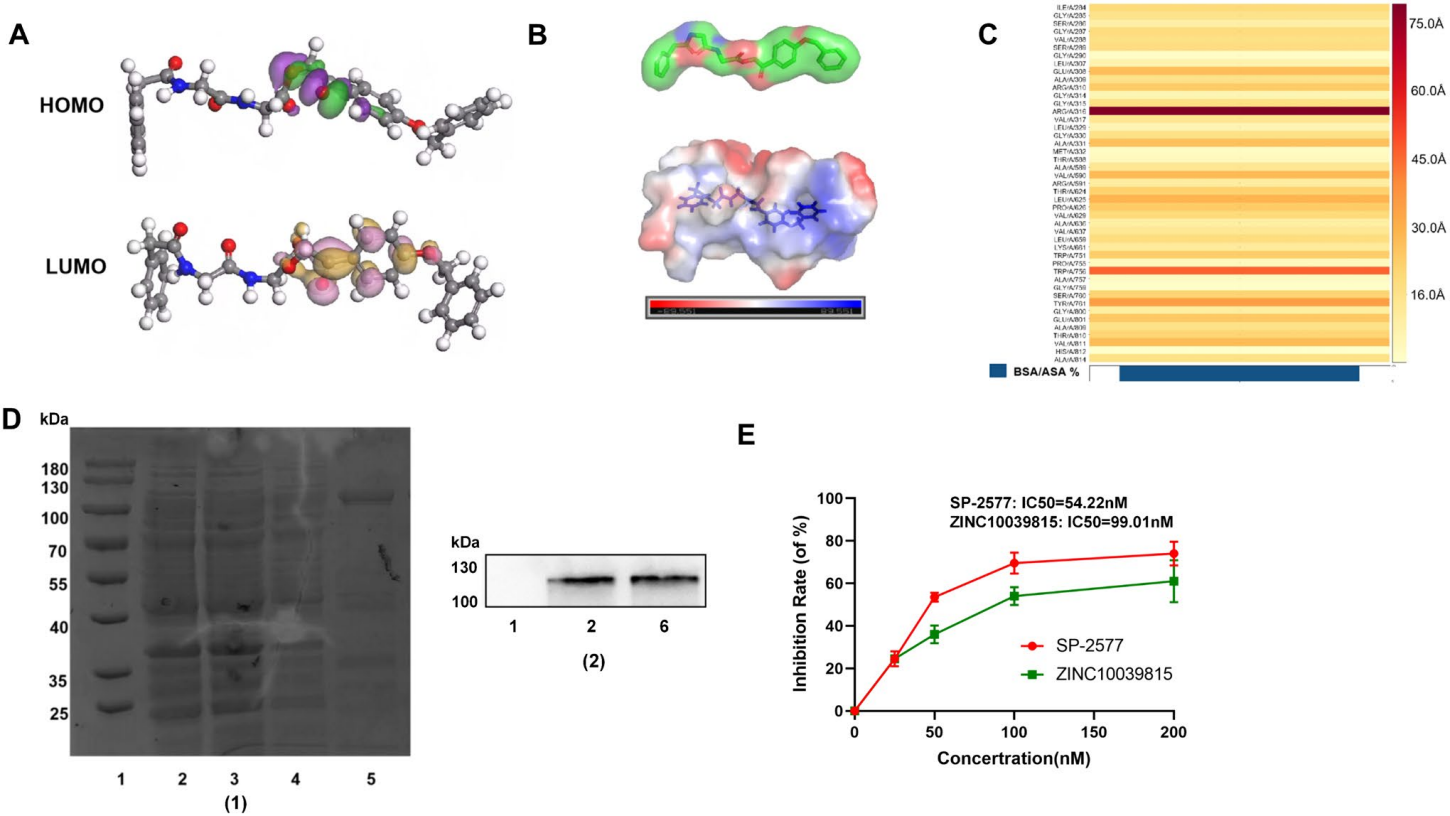
Analysis of inhibitory effect of ZINC10039815 on LSD1 protein and results of protein purification. **A** HOMO–LUMO structures of ZINC10039815. **B** Electrostatic potential diagram of the ZINC10039815 and its binding pocket with LSD1. **C** Buried surface area (BSA) of ZINC10039815. **D** (1) Results of protein purification: SDS-PAGE detection. 1: Marker. 2: Bacterial lysate. 3: Effluent from protein clarification liquid. 4: Wash buffer. 5: Elution buffer. (2) Western blot verification of the purified protein. 1: Maker. 2: Bacterial lysate. 6: Purified LSD1. **E** Inhibition curve of ZINC10039815 and SP-2577 against LSD1

The SMILES of ZINC10039815 are as follows: O=C(CNC(=O)Cc1ccccc1)NCC(=O)OCC(=O)c1ccc(OCc2ccccc2)cc1. The molecular mass is 474.513, and the logP is 2.467. The HOMO orbital electron density is located in the ethoxybenzene portion, and the LUMO electron density is located in the acetate-2-oxygen propyl ester. The coincident acetoyl portion of the two may have higher chemical reactivity (Fig. 5A). Figure 5B shows that ZINC10039815 has good electrostatic compatibility with the LSD1 protein, and charge dipole interaction is formed between them, suggesting potential inhibitory activity. Intermolecular interactions play a vital role in the stability of protein–ligand complexes and can be modeled by considering the physicochemical properties and complementarities of the binding interface, shape, and BSA evaluation is a useful approach. Therefore, the BSA was calculated for the complex of ZINC10039815 and LSD1 protein. The results showed that ZINC10039815 significantly buried the protein targeting LSD1 by 80%, particularly strongly burying the amino acid residues ARG_316 (55 Å) and TRP_756 (45 Å), indicating a strong binding affinity (Table 2). Consequently, ZINC10039815 is a promising LSD1 inhibitor that warrants further investigation.

**Table 2:** Compound ZINC1003981

Compound structure	
Molar Mass	474.513
logP	2.467
HOMO orbital	Ethoxybenzene portion
LUMO orbital	Acetate-2-oxygen propyl ester
BSA/ASA of LSD1 and ZINC10039815	80%
The main residue in BSA	ARG_316 TRP_756

### Compound inhibits the activity of LSD1

Based on the calculation method mentioned earlier, ZINC10039815 appears to exhibit a certain inhibitory effect on the activity of the LSD1 protein. To confirm this hypothesis, we conducted experiments using purified LSD1 protein (1.5 mg/ml) to assess the inhibitory activity of ZINC10039815 against LSD1. The results demonstrated a significant inhibitory effect, with a maximum median inhibitory concentration (IC_50_) of 99.01 nM (Fig. 5D, E). As a result, ZINC10039815 holds promise as a potential LSD1 inhibitor, offering a new chemical scaffold for the rational design of drugs targeting LSD1. Further research and optimization of this compound may pave the way for the development of effective treatments for LSD1-related diseases.

## Discussion

LSD1 plays a crucial role in the epigenetic regulation of cancer, and its inhibition has shown promising results in impeding cancer cell differentiation, proliferation, migration, and invasion. Consequently, anti-tumor drugs and therapies targeting LSD1 have emerged as a focal point in anti-cancer treatment. A plethora of LSD1 inhibitors, such as synthetic compounds, peptides, and natural products, have been reported. However, the long-term use of covalent inhibitors can lead to diverse adverse reactions due to their lack of specificity, while reversible inhibitors may result in off-target toxicity. Additionally, the cellular entry mechanism of polypeptide inhibitors remains to be elucidated (Noce et al. 2023). Thus, identifying a more selective and safer LSD1 inhibitor poses a novel challenge for cancer treatment.

In this study, we selected compounds from the ZINC15 database based on drug similarity to currently published reversible inhibitors of LSD1, aiming to identify potential compounds with efficacy as LSD1 inhibitors. The screening of potential LSD1-inhibiting compounds was carried out using computer-aided drug design. Furthermore, we confirmed the interaction between LSD1 and the selected compounds through molecular dynamics simulations using MM/PBSA. The results demonstrated that compound ZINC10039815 exhibited favorable docking fraction and binding free energy with LSD1. Notably, a stable interaction was observed between ZINC10039815 and key LSD1 residues, namely ARG_316 and TRP_756. Subsequent biochemical experiments confirmed the significant inhibitory activity of ZINC10039815 on LSD1 protein, with an IC50 value of 99.01 nM.

However, ADMET predictions raised concerns about the potential hepatotoxicity of ZINC10039815, necessitating further experimental studies to thoroughly assess its safety profile. Despite this issue, the inhibitory effect of ZINC10039815 on LSD1 protein opens new possibilities for the development of LSD1 inhibitors. Nonetheless, further research is essential to gain deeper insights into its specific therapeutic effects and to address any potential safety concerns. Overall, our study contributes valuable information to the search for novel and effective LSD1 inhibitors, and highlights the significance of ongoing research in this field to advance anti-cancer treatments. By overcoming the limitations of current LSD1 inhibitors, we can potentially pave the way for the development of more targeted and safer therapies for cancer treatment.

## Data Availability

The data are available from the corresponding author on reasonable request.
